# Implementing revised RED approaches to immunize in an evolving African landscape

**DOI:** 10.11604/pamj.supp.2017.27.3.11627

**Published:** 2017-06-21

**Authors:** Craig Alexander Burgess

**Affiliations:** 1 John Snow Inc, JSI research and Training Institute Inc, USA

**Keywords:** Immunization, immunization systems, Africa, Expanded Program on Immunization

## Commentary

Over the last 40 years, Africa’s immunization programs have averted many diseases, contributed to improving child mortality and, with a return on investment of more than 16 [1], helped strengthen African economies. Delivering cost effective health interventions, such as immunization, equitably and sustainably in future will require continued political and financial commitment and recognition of the need for strong health systems. This commitment and recognition are shown, on paper, in the 2016 African Ministers’ immunization conference, the 2030 Sustainable Development Goals (SDGs), the emerging Universal Health Coverage (UHC) agenda and the 2020 Global Vaccine Action Plan (GVAP).

However, global and regional level meetings and plans often miss the nuances of ‘how’ to practically strengthen systems to deliver immunization, as these discussions rarely discuss messy complex ground realities. In April 2016, the World Health Organization’s (WHO) Strategic Advisory Group of Experts on immunization (SAGE) recognized the need for more evidence in the ‘Science of Delivery’ [2]. African leaders, committed to delivering immunization and other cost effective health interventions to those who need them the most, will need better evidence of what works and what does not and will need to build upon initiatives that already have commitment and ownership.

There are various reviews of why children are not immunized and what strengthens immunization systems [3-6]. One immunization system strengthening initiative is the comprehensive Reach Every District (RED) approach, which has been implemented since the early 2000’s in many African countries. Africa’s regional DTP3 coverage increased from 52%, in 2000, to 76%, in 2015. RED’s five strategies are operationally focused, target sub-district levels and include i) planning and managing resources, ii) reaching target populations, iii) using data for action, iv) engaging with communities and v) supportive supervision practices. However, approaches such as RED will need to consider three emerging issues that will affect delivery systems in the next 20 years: urban growth, the need to reach more with more; and how to pay.

### Urban growth

By 2030, two thirds of the world’s population will be living in urban settings, with 90% of this urban growth occurring in Low and Middle Income countries [7]. Six of the ten countries with the highest urbanization rates are currently in Sub Saharan Africa. One third of urban populations live in poverty and children of rural-urban migrants are less likely to be fully immunized than urban non-migrants [8]. Immunization delivery models are over forty years old and these have traditionally focused on delivering to rural populations who face geographical barriers to access services. The old models have not been adjusted to meet the needs of the growing urban poor population, who face more complex social, cultural and financial barriers to service access and utilization. New models will need to take into consideration:

***High overall urban immunization coverage may mask disparities within urban populations, increasing the risk for disease transmission, epidemics and pandemics***

Urban populations are more complex, mobile, heterogeneous and less socially cohesive than rural populations. Children living in more densely populated areas are at increased risk of disease transmission and have a younger average age of first infection, when compared to their rural cousins. Accurately estimating target populations in urban settings is crucial for planning and implementing immunization programs. However, this is difficult because the private sector is weakly regulated, tracking records is harder and there may be little disaggregation of data to monitor slum areas. Urban specific delivery plans targeting urban poor could include equity analyses, microplanning, using data for action and targeted resource allocation. The underlying reasons for urban populations being socially marginalized are complex and include disparities in income levels, gender, education, ethnicity and access to clean water and adequate sanitation;

***Urban delivery and governance mechanisms are more complex:*** public health and municipal services are often complemented by unregulated private providers and NGOs, making coordination difficult. There are often unclear or overlapping levels of authority between different entities, making accountability harder;

***Lack of political will to increase urban poor visibility and predominant focus on curative care:*** the needs of poor urban populations often go unmet as they live in informal quasi-legal settlements which are often invisible to official statistics, population surveys, researchers and city planners. This invisibility due to vulnerability often leads to further exclusion in urban planning processes;

***Multiple program and multi-sector approaches are needed to address the underlying reasons for inequities:*** this requires extensive coordination and political and financial incentives for broad thinking stakeholders from different programs and sectors to rally around the needs of communities.

Strengthening systems in rapidly changing urban settings could benefit from social mapping, community engagement, and integrated approaches overseen by local multi-sector committees to address underlying socio economic determinants of ill health and poor access and utilization. If adapted to tackle some of these barriers in urban settings, the RED approach has potential to address access and utilization issues in urban settings.

### Delivering more to more

The GVAP, SDGs, UHC agenda and WHO’s framework on people-centered services all argue (on the basis of equity, sustainability, efficiency and effectiveness) for approaches that are based on integrated primary healthcare, driven by community needs. The May 2016 World Health Assembly passed a resolution on people centered services [9] that helps provide a framework for countries to consider aspects of integrated services. This approach may challenge those supporting African immunization delivery platforms to go beyond silo single disease program mentalities and look more closely at community needs. People / community centered approaches do not need to be in conflict with disease control approaches and implementing the RED approach has potential to reduce this frequent tension.

***Integrated approaches:*** immunization programs reach more beneficiaries than most other health programs. Vaccination schedules provide regular planned opportunities for contact with health systems and each scheduled contact between a vaccine recipient and the health system presents an opportunity to deliver other primary healthcare interventions at the same time. Avoiding missed opportunities requires integrated planning and implementation to increase system efficiency and effectiveness. However this take time consuming coordination and compromise between sectors and programs, overseen by politically strong and well financed holistic health policy planning groups. Without a push from the UHC agenda to provide an incentive for strong leadership at all levels or financial incentives to integrate at local levels, opportunities will continue to be missed. The RED approach has the potential to demonstrate how a successful delivery mechanism can be further strengthened to reach underserved populations in an integrated way.

***Reaching underserved communities:*** to maximize impact and return on investment, cost effective health interventions (such as immunization) need to reach populations whose needs are greatest. These populations are often the most marginalized, living in poverty with the highest disease burden including communities such as nomads, those displaced by conflict, urban poor and migrants. There is a need to understand communities, and this is one of the core elements of the RED approach. Consideration of activities such as social mapping, equity analysis and community and civil society engagement can increase the chances of services being acceptable, sustainable and appropriate. RED can also strengthen decision making processes to optimize immunization delivery modalities; such as routine immunization sessions provided at fixed site, outreach, child health days, in schools or campaign (in areas with weak Government infrastructure or when there is a need to have rapid impact).

***Immunization beyond infancy using a life cycle approach:*** immunization services deliver multiple vaccines often requiring varying schedules and booster doses. Immunization itself affects the age distribution of diseases. Immunization schedules now need to go well beyond the basic immunization schedules of pregnancy and infancy. To maximize health benefits and return on investments, health systems will need to deliver immunization services to more African infants, pregnant mothers, school children, adolescents and vulnerable adults. This requires a change in thinking beyond traditional schedules and, in the context of a primary healthcare approach, be more aligned to a life cycle or continuum of life approach to delivery.

### Paying for it all

New vaccines are more expensive than traditional vaccines, even when subsidized by Gavi, the Vaccine Alliance. More countries are defaulting on Gavi co-financing payments for new vaccines and some countries are struggling even to afford traditional (non-Gavi funded) vaccines, without support from UNICEF or other partners [10]. More African countries will enter into Middle or High Income status and graduate from Gavi support in the coming years. This should be celebrated from a development perspective, but financial support for vaccines and their delivery costs will increasingly need to be met from scarce domestic revenues. Sustainability is an issue and it is crucial that the budgets for all vaccines and delivery costs are transparently reflected ‘on budget’ to ministries of finance.

Disease control initiatives such as polio eradication and measles elimination have paid for many immunizations related costs, mainly campaigns and surveillance in African countries. Polarization of viewpoints between top down, vertical disease control programs and bottom up, primary health care systems strengthening horizontal approaches are well known and need to be bridged with compromise. Focus on externally driven and funded campaigns may have negative effects on long term financial sustainability and ownership of immunization programs; staff compensation for disease control programs may be more than national norms and focus may be more on quality campaigns and surveillance of one disease, rather than broader systems issues. Once disease specific goals such as polio eradication are achieved, financial resources decrease sharply as they are tied to disease goals. Eradication of polio from the African continent is stimulating debate about polio asset transition, which is not simply a ‘no brainer’ [11] of transferring assets from one disease control initiative to another. The transition of polio eradication assets requires careful thought from multiple stakeholders with broad perspectives to ensure polio assets can support immunization delivery platforms to sustainably deliver multiple antigens and other cost effective MNCH interventions. Priorities should be based on the country context and local needs, using a bottom up approach. This is where the RED approach emphasizes community, health facility and district teams to manage and budget resources effectively.

Ministries of Health and Finance could consider ways of diversifying and protecting income streams for immunization programs. Options include a mixture of Social Health Insurance (as countries consider adopting Universal Health Coverage), making use of the Global Finance Facility (as part of holistic approach to MNCH approaches), funding vaccines on a regional basis using a PAHO-like revolving fund model (where countries join together to bulk purchase vaccines) and earmarked taxes (such as sin taxes on tobacco and alcohol).

***A possible practical solution: ‘Seeing red’ to revise and implement RED approaches***

WHO AFRO, together with UNICEF, USAID, MCSP/USAID, John Snow Inc. (JSI), Bill and Melinda Gates Foundation (BMGF), US Centers for Disease Control (CDC) and other partners, is currently supporting the revision of the 2008 RED guidelines to take into consideration the challenges of urbanization, integration, community engagement, life cycle and financial sustainability.

Learning lessons from implementing context-specific RED approaches in Africa has potential to generate more evidence for policy makers who need to know what works and what does not for strengthening immunization systems. Renewed political and financial commitment for countries to roll out and integrate context-specific RED approaches into health programs could play a central role in strengthening underlying immunization systems to deliver interventions more effectively, equitably, efficiently and sustainably. With its operational approach and explicit links to community needs, applying the RED approach has much potential not only to contribute to countries immunization plans, but also overall UHC and development plans.

## Authors’ contributions

The author declares no competing interest.
